# Malignant Catarrhal Fever: Understanding Molecular Diagnostics in Context of Epidemiology

**DOI:** 10.3390/ijms12106881

**Published:** 2011-10-18

**Authors:** Hong Li, Cristina W. Cunha, Naomi S. Taus

**Affiliations:** 1 Animal Disease Research Unit, USDA-Agricultural Research Service, Washington State University, Pullman, WA 99164, USA; E-Mails: cwcunha@vetmed.wsu.edu (C.W.C.); tausns@vetmed.wsu.edu (N.S.T.); 2 Department of Veterinary Microbiology and Pathology, Washington State University, Pullman, WA 99164, USA

**Keywords:** malignant catarrhal fever, diagnosis, cELISA, PCR, gammaherpesvirus, infection

## Abstract

Malignant catarrhal fever (MCF) is a frequently fatal disease, primarily of ruminants, caused by a group of gammaherpesviruses. Due to complexities of pathogenesis and epidemiology in various species, which are either clinically-susceptible or reservoir hosts, veterinary clinicians face significant challenges in laboratory diagnostics. The recent development of specific assays for viral DNA and antibodies has expanded and improved the inventory of laboratory tests and opened new opportunities for use of MCF diagnostics. Issues related to understanding and implementing appropriate assays for specific diagnostic needs must be addressed in order to take advantage of molecular diagnostics in the laboratory.

## 1. Introduction

Malignant catarrhal fever (MCF) is a clinically dramatic and often lethal infection of many species of Bovidae and Cervidae [1–3] caused by a member of the MCF virus (MCFV) group [2,4] that belongs to the genus *Macavirus* in the subfamily Gammaherpesvirinae [2,4]; these viruses exist in nature as inapparent infections in well adapted hosts. Currently, 10 members within the MCFV group have been identified, and six of them are clearly associated with clinical disease [4]. Alcelaphine herpesvirus 1 (AlHV-1) and ovine herpesvirus 2 (OvHV-2) are the major causative agents responsible for wildebeest-associated MCF (WA-MCF) and sheep-associated MCF (SA-MCF), respectively. AlHV-1 is endemic in wildebeest, in which it is a subclinical infection [5]. Domestic and wild sheep are reservoirs for OvHV-2 [3]. Other MCFVs known to cause disease include caprine herpesvirus-2 (CpHV-2), which is endemic in goats [6,7], an MCFV of unknown origin causing disease in white-tailed deer (MCFV-WTD) [8,9], ibex MCFV (MCFV-ibex) carried by ibex [10], and an AlHV-2-like virus carried by Jackson hartebeest [11]. The remaining four viruses carried by roan antelope [12], oryx, muskox [10], and aoudad [4] have not yet been associated with disease. MCF is increasingly being recognized as the cause of significant economic losses in several major ruminant species [13–15], as well as a threat to certain other susceptible species held in mixed-species confinement [16–18]. Due to the complexities of pathogenesis and its epidemiology, clinicians and veterinarians face significant challenges in diagnosing MCFV infection and/or disease. However, recently developed molecular diagnostic assays have improved the detection and differentiation of MCF causative viruses, and increased accuracy of laboratory assays in confirming MCFV infection and/or disease in various species. Understanding the appropriate application of newly developed MCFV diagnostic assays for each epidemiological situation is necessary to take advantage of these molecular diagnostics. The situational application of MCFV diagnostics in veterinary diagnostic laboratories is the focus of this mini review.

## 2. Infection, Disease and Clinical Epidemiology

Malignant catarrhal fever occurs in clinically susceptible hosts, such as cattle, bison, deer and pigs when a sufficient dose of an MCFV is transmitted from a reservoir host. Disease usually has an acute onset and involves a spectrum of symptoms that may include corneal opacity, profuse ocular and nasal discharge, diarrhea, enlarged lymph nodes, fever and anorexia. The distribution of lesions differs slightly depending upon the species affected but the basic pathological features are consistent and include widespread lymphoproliferation, vasculitis, and epithelial necrosis [19–23]. The transmitting viral dose does not affect lesion severity once clinical MCF develops [24]; however, transmitting viral dose is significantly correlated with the incubation period and the timing of first viral DNA detection by polymerase chain reaction (PCR) in peripheral blood leukocytes (PBL) [25]. Both clinical presentation and pathological features are of significant diagnostic value [21]. Detection of viral DNA by PCR in PBL and tissues, especially at high levels, can support the diagnosis of MCFV caused disease [26].

Experimental studies in cattle, bison and sheep indicate that the susceptibility of various ruminant species to OvHV-2 infection and MCF varies significantly. Bison are approximately 1000 times more susceptible to clinical MCF than cattle [14,27]. The difference in susceptibility to MCF between bison and domestic sheep is more than six orders of magnitude [24,28]. Although MCF is usually fatal once clinical signs develop, especially in bison, cattle and certain species of deer, subclinical infection can occur. Subclinical infections with an MCF group virus in bison and other species, such as deer and cattle, have been documented [29–31]. For instance, a prospective study using 300 healthy bison showed that 23.7% of the bison (71/300) were antibody-positive for MCF group viruses and 11.3% (8/71) of the antibody-positive animals had detectable OvHV-2 DNA in the peripheral blood by PCR [21]. Recent experimental infection of cattle and bison with OvHV-2 by aerosol transmission further confirms that clinically susceptible hosts can be subclinically infected [24,27].

MCF may occur wherever a reservoir host is present and there are clinically susceptible animals in close proximity. The epidemiology of MCF, regarding the pattern of virus transmission from reservoir hosts to clinically susceptible hosts, has been relatively well defined for AlHV-1 and OvHV-2, the two major MCFVs [1–3]. Both viruses are shed into the environment via nasal, and perhaps ocular, secretions from their reservoirs [32,33]. Clinically-susceptible species acquire the virus through inhalation, although ingestion of virus-laden secretions from contaminated foodstuffs or water has also been suggested as a route of transmission [34]. Efficient transmission via infected secretions is enhanced by close contact and by a cool, moist environment; however, long distance transmission has been documented [35]. MCFV is not transmitted by natural means from one clinically-susceptible host to another; affected animals are dead-end hosts [2,14,36]. Virtually all reservoir hosts are infected with their own distinct MCFV; however, a dual infection can occur under certain conditions [6]. The infection in reservoir hosts is usually subclinical, although MCF-like disease has been rarely reported in sheep and goats [28,37].

The epidemiology of AlHV-1 and OvHV-2 within their natural hosts has been relatively well defined, and differs significantly from each other [1,2]. The epidemiology within the wildebeest species involves both horizontal and vertical transmission. A portion of wildebeest calves are born infected through the transplacental route; however, most calves are infected horizontally from previously infected cohorts. Intense viral shedding from the wildebeest occurs predominantly during the first 90 days of life through ocular and nasal secretions [32,38]. Neutralizing antibody develops by about 3 months of age, after which viral shedding declines dramatically [32]. Adult wildebeest shed a relatively low level of the virus, except during periods of stress or parturition [38,39]. Wildebeest-associated MCF occurs seasonally with wildebeest calving [40], and the virus originates from the wildebeest calves up to the age of about 4 months [32,41].

The epidemiology of OvHV-2 within sheep has become better understood. Although lambs can be infected at an early age [42], similar to wildebeest calves, the majority of lambs are not infected until after 2 months of age, under natural flock conditions [43]. If lambs are removed from contact with infected sheep prior to that age, they remain uninfected and can be raised virus free [44]. This knowledge is being used by sheep producers and zoos [16] to produce OvHV-2-free sheep. Data support the concept of delayed, rather than congenital or perinatal, infection of lambs with OvHV-2. The delayed infection in lambs is largely due to the viral dose at first exposure [45], rather than age-related susceptibility or passive-immune protection [46]. Both adolescent lambs and adult sheep shed virus predominantly though nasal secretions [33]. Lambs between 6 and 9 months of age shed virus more frequently and intensively than at any other stage of life. No correlation between parturition and virus shedding levels in adult sheep has been found [33], suggesting that the likelihood of transmission from adult sheep is relatively stable and low year-round; therefore, the small increase of SA-MCF in spring during lambing season could reflect factors other than viral shedding levels, such as climate conditions and seasonal variations in stock densities that could influence exposure intensity.

Little is known concerning epidemiology of other viruses in the MCFV group. Based on phylogenetic analysis of a portion of the DNA polymerase gene that is relatively conserved among herpesviruses [4], all MCFVs identified to date can be clustered into two major groups: (1) the Alcelaphinae/Hippotraginae group, which includes AlHV-1, AlHV-2, hippotragine herpesvirus 1 (HiHV-1), and the MCFV carried by oryx; and 2) the Caprinae group including OvHV-2, CpHV-2, MCFV-WTD, and the MCFVs carried by ibex, muskox, and aoudad [4]. These data suggest the epidemiology of these viruses within the groups may be similar. The viruses within the group share certain biological properties; for example, the viruses in the Alcelaphinae/Hippotraginae group can propagate in cell culture, and a recent study on CpHV-2 transmission among goats showed that CpHV-2 has a similar transmission pattern as OvHV-2 [47].

It is important to note that naturally occurring MCF in multiple-species mixed operations, such as zoos, state fairs, wildlife parks and game farms, is linked to the reservoir hosts: goats have been responsible for CpHV-2 induced MCF in sika deer [48,49], also white-tailed deer [50], and pronghorn antelopes [51]; and ibex have been responsible for several cases of MCF in bongo [52] and an anoa [53]. With the complexity of multiple-species environments, confirmation of MCFV caused disease and differentiation of a causative virus not only requires accurate diagnostic tools, but also epidemiological information.

## 3. Serological Tests

Several serological assays have been developed for detection of antibodies against MCFVs, and all the assays use the alcelaphine herpesviruses as antigens, predominantly AlHV-1, because these viruses can be propagated *in vitro*. These assays include virus neutralization (VN), immunoblotting, enzyme-linked immunosorbent assay (ELISA)/competitive-inhibition ELISA (cELISA), immunofluorescence assay (IFA)/immunoperoxidase test (IPT), and complement fixation test [54–60]. These tests can be divided into three categories: neutralizing antibody-, polyclonal antibody- and monoclonal antibody-based assays. Viral neutralization tests have been developed for detection of antibodies to AlHV-1 or other viruses in the *Alcelaphinae/Hippotraginae* group of both reservoir and clinically-affected hosts [61]. The VN tests are highly specific and work well for detection of infected wildebeest or other related hosts, such as hartebeest and topi. Infected sheep usually develop no or low neutralizing antibody responses to AlHV-1 [62]; therefore, the viral neutralization test is of very limited use in detection of antibodies in animals infected with OvHV-2 or the other related viruses carried by *Caprinae* species. Polyclonal antibody-based assays, including ELISA, IFA, and IPT among others, detect antibodies against multiple epitopes of AlHV-1. Generally, these tests have good sensitivity, but reduced specificity, due to cross-reactivity with other herpesviruses, such as bovine herpesviruses 1 and 4 [63,64]. To increase specificity, a cELISA for detection of MCF viral antibody was developed using an antibody (15-A) against an epitope conserved among all MCFVs examined to date [58]. The MCF cELISA has high specificity and sensitivity due to the use of the monoclonal antibody and its direct conjugation with the detecting enzyme [65]. A recent study reported an ELISA using AlHV-1 antigens that resulted in good agreement with the cELISA using a set of field bovine serum samples [66]. However, the cELISA still offers the advantage of testing samples from many species without the need for species-specific enzyme-labeled conjugates for each species being tested [65]. In addition, relatively crude antigens may be used in the cELISA without reducing the desired specificity.

Serological tests are best used for surveying asymptomatic animals in the field and a positive result is indicative of infection. Virtually all reservoir hosts of MCFVs are infected and consistently develop antibodies, which can be detected by any of the serological tests. Uninfected lambs under 4 months of age may be antibody-positive due to the presence of maternal antibody [43]; therefore, a serological test should not be used to determine the infection status of a young animal, especially for the production of MCFV-free animals for mixed-species programs [44]. It is unusual for adult reservoir hosts such as sheep and goats to be seronegative. However, young animals less than 12 months of age, especially in a small flock or herd, may test antibody negative due to exposure to a low collective virus dose shed from infected animals [45]. An animal challenged with a low dose of virus may take more than 4 weeks to become seropositive [67], which should be considered when serology is used to test an animal in pre-shipment or quarantine procedures. Additionally, animals from specially-designed programs intended to produce MCF-free animals, or from zoos, small operations, or other environments where the animals are separated at an early age from an infected flock or herd and hand raised are expected to be seronegative to MCF antibodies. Overall, serology is reliable for determining infection status in adult reservoir hosts, although it does not differentiate MCFVs. Detection of MCF viral antibodies in clinically susceptible species, such as cattle, bison and deer, also indicates infection. However, since a significant percentage of these species can be subclincially infected with the virus [21,29,30], the presence of antibody supports the diagnosis of disease only when associated with histopathological evidence suggestive of MCF.

## 4. Polymerase Chain Reaction (PCR) Assays

PCR has become an important tool in MCF molecular diagnostics. The first PCR for detection of AlHV-1 DNA was reported in 1990 [68] and since then, at least a dozen PCRs in different formats have been developed targeting various MCFVs [6,8,69–77]. Among those, the one with the most significant impact is the PCR specific for detection of OvHV-2 DNA developed by Baxter *et al*. [70]. This assay in nested format was developed from the base sequence of a fragment cloned from a lymphoblastoid cell line that was derived from an acute case of SA-MCF. The primers target a DNA fragment in the ORF 75 of OvHV-2, a gene coding for a viral tegument protein [70]. The specificity for OvHV-2 arises from one of the primers (#556), which binds to a region of low homology between OvHV-2 and AlHV-1 [70]. This nested PCR has high sensitivity and is validated for detection of OvHV-2 DNA in infected sheep as well as in animals with clinical MCF [64,78]. The assay has been widely used in veterinary diagnostic laboratories; however, its use as a routine method to detect OvHV-2 DNA for confirmation of clinical SA-MCF in diagnostic laboratories may be problematic due to a high potential for amplicon contamination leading to false positive results in diagnostic laboratories. The second important step in MCF PCR development was a quantitative PCR (qPCR) for OvHV-2 DNA developed by Hussy and coworkers [77]. The OvHV-2-specific primer-probe set for the real-time qPCR is based on the same sequence of genomic OvHV-2 DNA that had been used for the nested PCR (ORF 75 gene) [77] and showed adequate sensitivity for clinical samples [73]. Another significant advance in MCF molecular diagnostics, especially for mixed-species operations, such as zoos, wildlife parks, and game farms, was the development of a multiplex PCR for detection and differentiation of MCFVs known to cause disease [76]. The multiplex PCR is a probe-based real-time PCR that targets a polymorphic region in the viral DNA polymerase gene containing unique sequences for each pathogenic MCFV of interest [76], and represents a rapid, reliable, and differential method for the identification of MCFVs in clinical samples.

These newly developed assays have significantly improved MCF diagnostics at the molecular level and the key question for clinicians and veterinarians is: which assay should be used? In MCF diagnostics, it is important to first consider epidemiological information. In most cases, it is clear whether the disease is associated with sheep, wildebeest, or another reservoir host, and a test specific for the expected virus can be employed. However, when a sample comes from a zoo or game farm where various reservoir hosts may have been in contact with the clinically susceptible species, multiplex PCR or several PCRs specific for different individual viruses should be considered. In general, all samples can be divided into two large categories: clinical cases (whether or not the disease is caused by an MCFV) and subclinical cases (whether or not an animal is infected with an MCFV). Both clinical and subclinical MCFV infections occur in clinically-susceptible hosts, such as cattle, bison, and deer. In clinical cases of SA-MCF, levels of OvHV-2 DNA in PBL and tissues are usually high, ranging from a thousand to over a million copies per microgram DNA, which can be easily detected by the OvHV-2 qPCR [24]. The qPCR is highly recommended for diagnosis of OvHV-2-induced MCF. In clinical samples derived from mixed-species operations, the multiplex PCR is recommended to confirm which virus is causal [76]. In subclinically infected bison and cattle, levels of OvHV-2 DNA in PBL are low, and in most cases not detectable even by nested PCR [21]. The confirmation of infection in clinically susceptible hosts that are disease-free is usually an irrelevant issue, since the subclinical infection leading to clinical MCF in cattle and bison is uncommon, and transmission of the virus from an infected animal to its cohorts is unlikely [36,79]. Virtually all reservoir hosts, such as sheep, goats and wildebeest, are infected with their respective MCFVs and their infection status can be generally confirmed by serology. There are some instances where it is necessary to use PCR to document that animals are MCFV-free. For example, in order to raise OvHV-2-free sheep and perform an early separation of uninfected lambs from a positive flock, initial screening of lambs requires a PCR test [44]. In these cases, it is necessary to use a nested PCR to maximize sensitivity, since the nested PCR is more sensitive than the qPCR [26]. Veterinarians or managers in mixed-species operations usually want to know which MCFV(s) infect their reservoir species and request PCR for that identification. One should keep in mind that: (1) some PCR assays may not have adequate sensitivity to detect viral DNA in PBL of certain reservoir hosts; for example, CpHV-2 specific PCR detects only 85% of infected goats that are seropositive; and (2) viral DNA levels in PBL may vary among different reservoir species: infected sheep usually have enough viral DNA in their PBL to be detected by PCR, while viral DNA in PBL cannot be detected by PCR in most infected oryx [10] or black wildebeest [80]. One also should keep in mind that all infected reservoir hosts are considered to be the source for virus transmission regardless of which virus the animal carries. Although it is extremely rare, MCF can occur in reservoir hosts, which is supported by the experimental induction of MCF in sheep by high dose OvHV-2 challenge [28]. In these cases, antibody or PCR testing has little diagnostic value, and the verification of suspected cases of MCF in the reservoir species will require additional laboratory data (e.g. compatible histological lesions, and ruling out other differential diagnoses). It is necessary to keep in mind that an unidentified MCFV can be present in cases with strong clinical and pathological indications of MCF, even though all existing PCRs are negative. Although the amplification products require verification by sequencing, the degenerate PCR that pan-specifically targets the herpesviral DNA polymerase gene [81] is a useful tool to identify new members of the MCFV group, and will continue to be used in the MCF diagnostic field.

## 5. Other Potential Molecular Diagnostic Tests

In cases where MCF is suspected in a reservoir host, the use of a diagnostic assay directly targeting a viral component that is associated with lesion development would be of great relevance to confirm the diagnosis. The *in situ* PCR specific for OvHV-2 was initially thought to have diagnostic potential [82], but it was shown to be technically difficult for adaptation as a routine diagnostic tool. Recent studies showed that the OvHV-2 major capsid protein is detected in sheep lung during initial pulmonary viral replication [83] and in rabbit tissues with OvHV-2 induced MCF [84]. Further data showed that the OvHV-2 ORF 25 gene encoding the major capsid protein was highly expressed in tissues of bison with experimentally induced MCF, and levels of the transcripts were significantly co-related with lesion severity [85,86]. These data suggest that ORF 25 gene transcripts and/or the capsid protein can be a diagnostic target for OvHV-2 induced MCF. Monoclonal or monospecific polyclonal antibodies against the OvHV-2 capsid proteins can potentially be generated and used in an immunohistochemistry-based assay to provide a definitive confirmation of the disease by detecting viral proteins in tissues with lesions. Also, detection of the gene transcripts by reverse-transcriptase PCR (RT-PCR) can be an alternative assay. A preliminary study showed that transcripts of the ORF 25 gene can be detected in the RNA samples obtained from formalin-fixed, paraffin-embedded tissues [87], suggesting that real-time RT-PCR may have potential for clinical diagnosis of MCF.

## 6. Summary

Several newly developed molecular diagnostic assays are now available for MCF and due to the complexity of pathogenesis and its epidemiology in various species, including clinically-susceptible and reservoir hosts, the challenge for clinicians and veterinarians is to choose the right test for confirmation of the disease or infection. The broad range of natural hosts for MCFVs can be generally divided into two categories: reservoir hosts (such as sheep, goats, and wildebeest) and clinically-susceptible hosts (such as cattle, bison, and deer). Virtually all reservoir hosts are infected and serological assays are useful and efficient to determine infection status except for very young animals with maternal antibodies. Using PCR for detection and differentiation of MCFV in reservoir hosts is generally used only for very specific purposes; for example, nested PCR is needed to determine whether lambs are free of OvHV-2 in order to establish an OvHV-2-free flock. Serology and PCR have little value for confirming the disease in a reservoir host with suspected MCF. Both clinical disease and subclinical infection occur in clinically susceptible hosts. Both nested PCR and real-time PCR work well for detection of viral DNA in PBLs and tissues of animals with clinical MCF. The use of real-time qPCR is highly recommended for routine testing due to its adequate sensitivity, high specificity, and decreased vulnerability to cross contamination of amplicons. Serological testing, specifically the cELISA, is recommended for screening the infection status in clinically susceptible hosts rather than PCR, which may result in a high number of false negatives due to low levels of viral DNA in PBLs during subclinical infection. It is important to keep in mind that epidemiological information needs to be considered first in order to determine which specific PCR should be used to confirm the diagnosis of the cause of clinical disease. If samples are from mixed-species operations, the multiplex PCR is the first choice, because it saves significant time, labor and cost. In case where the multiplex PCR results are negative, the herpesvirus degenerate PCR would be the next option. With the advances in molecular technologies and better understanding of the disease, a new generation of tests for MCF with better sensitivity, specificity, and convenience may be developed in the near future.
